# Divergent Recovery Paths in Musicians’ Dystonia

**DOI:** 10.5334/tohm.1101

**Published:** 2026-01-20

**Authors:** Johanna Doll-Lee, Edoardo Passarotto, Eckart Altenmüller, André Lee

**Affiliations:** 1 Department of Neurology, Hanover Medical School, DE; 2 Institute of Music Physiology and Musicians’ Medicine, University for Music, Drama and Media, Hannover, DE; 3 Department of Neurosciences, University Padova, IT; 4 Department of Neurology, TUM Medical School Munich, DE

**Keywords:** Musician’s Dystonia, Embouchure Dystonia, Focal Hand Dystonia

## Abstract

**Background:**

Musician’s dystonia, characterized by a loss of voluntary motor control during performance, predominantly manifests as two forms: Musician’s Hand Dystonia (MHD) and embouchure dystonia (ED). ED involves more muscle groups compared to MHD, complicating diagnosis and treatment. Moreover, treatment options for MHD are either not viable for ED, such as botulinumtoxin injections, or less effective in ED, such as anticholinergic drugs.

**Methods:**

This study aimed to compare long-term subjective playing abilities at the onset of dystonia and after treatment between ED and MHD patients. We also evaluated the variation in subjective playing ability over time between the groups.

**Results:**

Findings revealed that playing ability at onset and the current playing ability are significantly lower in ED than in MHD. Notably, a significant improvement in playing ability over time was observed solely in MHD. Additionally, fewer ED patients sought therapeutic interventions, underscoring a scarcity of treatment options.

**Discussion:**

The results highlight a poorer prognosis for ED compared to MHD, likely due to the increased muscle involvement in ED and limited compensatory mechanisms available to these patients, emphasizing an urgent need for alternative therapeutic strategies for those affected by ED.

## Introduction

Musician’s dystonias (MD) is a highly disabling focal dystonia that leads to the loss of control of highly trained movements at the instrument [1]. The most common forms of MD are upper limb dystonia of the fingers (Musician’s Hand Dystonia, MHD) and embouchure dystonia (ED) [2,3]. Available treatment options for MD in general include anticholinergic drugs such as Trihexyphenidyl (THX), the injection of Botulinumtoxin (BTX) into affected muscles and Retraining, which involves a long-term systematic relearning of movements over several years. However, the intake of THX as well as retraining therapies have been shown to be less effective in ED patients [4]. Moreover, while BTX treatment is considered the standard and potentially most effective treatment of focal dystonias such as MD [4,5,6], it is not successfully applicable to most of the ED patients [2]. This is mainly because, while a skilled clinician can easily identify the affected fingers and their associated muscles in MHD, a number of muscles – including the orofacial, pharyngeal, jaw and tongue muscles – are involved in ED that are difficult to identify and to localize [2,3,7]. Although new real-time MRI-techniques allow visualisation of dysfunctional movements of the tongue and the oral cavity, it remains still difficult and risky to apply BTX to these muscles [8,9,10].

In view of these differences in the quantity or efficacy of treatment options, we were interested in investigating whether the degree of improvement in subjective playing ability differs between ED and MHD patients. To our knowledge, there is only one study comparing ED and MHD, which found that improvement was reported less often in ED patients compared with MHD patients [4]. Thus, the aim of the present study was to test the following hypotheses:

1. Within group (MHD or ED): Subjective playing ability is lower at dystonia onset than today (= when filling out the questionnaire).
2. Between groups: The change in subjective playing ability between after onset and today differs between groups.
3. The difference in subjective playing ability (today minus after onset) is greater for MHD than ED.

## Methods

### Participants

We included 265 patients (age [mean ± sd] 49 ± 11 years; 57 female [21%]; 208 male [78%]) with MHD and 92 patients (age 48 ± 11 years; 19 female [21%]; 73 male [79%]) with ED (Table 1A). With a questionnaire [4] we retrospectively asked the patients to assess their playing ability in % at two time-points: after dystonia onset and today (i.e. when filling out the questionnaire), where 100% was the playing ability before onset of dystonia. We furthermore calculated the difference in playing ability between today and after dystonia onset (today minus after onset), where a positive value signifies an improvement, zero no change and a negative value a deterioration.

**Table 1:** **Participants’ characteristics**.


*A) SUBJECT RELATED DATA*				

	MHD		ED	
	
	MEAN	SD	MEAN	SD

Age (yrs)	49	11	49	11

	n	%	n	%

**Gender**				

Female	57	22%	19	21%

Male	208	78%	73	79%

***B) INSTRUMENTAL GROUPS***				

	**MHD**		**ED**	
	
	**n**	**%**	**n**	**%**

**Instrumental Group**				

Keyboard- instrumentalists	94	35.5%	0	0%

plucked instrument	61	23.8%	0	0%

Woodwind- instrumentalists	60	22.6%	19	20%

String- instrumentalists	41	15.4%	0	0%

percussionists	5	1.9%	0	0%

Brass- instrumentalists	2	0.7%	73	80%

other	1	0.4%	0	0%


Abbreviations: yrs = years; SD = standard deviation; MHD = musician’s hand dystonia; ED = embouchure dystonia.

It is known that male gender, the amount of practice, pain or traumata in the history or a positive family history for neurological disorders are risk factors for developing MD [11,12,13,14,15,16,17]. We therefore asked for these parameters in the questionnaire. Patients were asked to estimate the number of hours they practiced per day during pre-defined periods of their training. Furthermore, we asked which therapies (BTX, THX, retraining) participants had received so far and calculated the percentage of participants having received one or more of these therapies for both groups.

The study was approved by the local ethics committee (vote 2163-2014). All participants gave written consent according to the Declaration of Helsinki.

### Statistics

We applied a 2-way-ANOVA with group (MHD, ED) and time (after dystonia onset, today) as independent variables and the playing ability as dependent variable. We then applied the Tukey post-hoc test.

To test for the difference in playing ability between groups (ED/MHD) we applied a t-test with group as independent variable and difference in playing ability as dependent variable.

To test for differences between groups with regard to gender distribution, patients with pain/trauma in the past, positive family history for neurological disorders and the number of patients having received treatment we applied a χ^2^-test.

To test for differences between the groups with regard to cumulated practice time, practice time per year, age when beginning to play the instrument, time between beginning to play the instrument and dystonia onset and age at dystonia onset, we applied Wilcoxon rank sum tests. A family wise error correction was applied for multiple testing.

## Results

In the MHD group, 94 patients (35.5%) played a keyboard instrument, 61 (23.8%) played a plucked instrument, 60 (22.6%) played a woodwind instrument, 41 (15.4%) played a string instrument, five (2%) played a percussion instrument, two (1%) played a brass instrument, and one (0.3%) played the bagpipe. In the ED group, 73 patients (79.3%) were brass instrumentalists and 19 (20%) were woodwind instrumentalists (Table 1B).

There were no differences between groups with regard to gender distribution (χ^2^ = 0.03, df = 1, p = 0.9); number of patients with pain/trauma in the history (χ^2^ = 1.2, df = 1, p = 0.2) and positive family history (χ^2^ = 2.4, df = 1, p = 0.12). However, fewer patients with ED had received treatment (χ^2^ = 24.7.5, df = 1, p << 0.001). No differences were found with regard to cumulated practice time (W = 10304, p = 0.9), mean practice time per year until dystonia onset (W = 10597, p = 0.6) or time between beginning to play the instrument and dystonia onset (W = 11528, p = 0.4) or age at dystonia onset (W = 10951.5, p = 0.14) (Table 2). However, ED patients had started instrumental training significantly later (W = 10407, p = 0.03).

**Table 2:** Comparisons between groups.


	MHD	ED		

	%		χ^2^	p-adj

gender distribution	see Table 1	see Table 1	0.03	0.9

patients with pain/trauma	22%	27%	1.2	0.2

positive family history	15%	9%	2.4	0.12

received treatment	80%	53%	24.7	<<0.001

	median		W	P

cumulated practice time (hrs)	22554	27774	10304	0.9

practice time per year until onset (hrs/year)	1004	1006	10597	0.6

time (beginning to play – onset) (yrs)	24	26	11528	0.4

age at dystonia onset (yrs)	34	37	10951	0.1

age when beginning to play the instrument (yrs)	9	9.5	10407	0.03


Abbreviations: yrs = years; MHD = musician’s hand dystonia; ED = embouchure dystonia. p-adj: false discovery rate -adjusted p-values for multiple testing.

**Figure 1:**
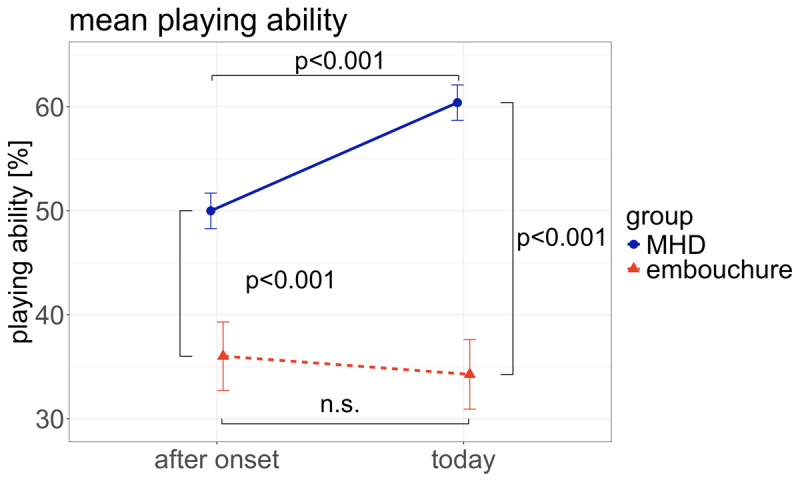
**Playing ability after dystonia onset and today for musician’s hand dystonia (MHD) and embouchure dystonia (ED)**. In ED, playing ability is significantly lower than in MHD at onset as well as today. Whereas in ED no significant change in playing ability occurs, there is a significant improvement in MHD. Error bars indicate standard error.

The ANOVA revealed a main effect for time (after onset/today, df = 1, F = 16.6, p≪0.001) and group (MHD/ED, df = 1, F = 15.5, p≪0.001). Moreiver, there was an interaction effect (df = 1, F = 5.7, p = 0.05). Tukey post hoc test revealed a significant difference between the playing ability after onset and today for the MHD group (p < 0.001), who improved over time, but not for the ED group (p = 1) (Figure 1). A significant difference in playing ability between MHD and ED was found after onset of dystonia (p < 0.001) and today (p < 0.001), with the playing ability being significantly lower for the ED group (Figure 1).

**Figure 2:**
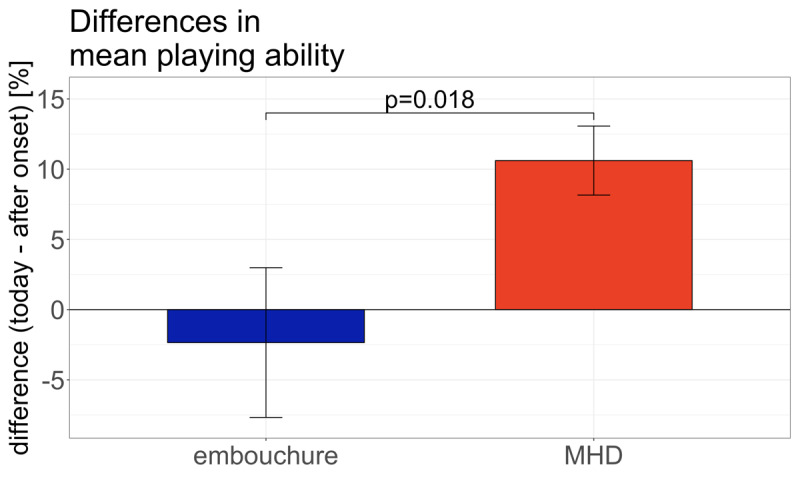
**Difference in mean playing ability between after onset and today for ED and MHD**. A negative value indicates a deterioration, a positive value an improvement since dystonia onset. There is no improvement in the ED group. Error bars indicate standard error.

The t-test revealed a significant difference in playing ability between groups (t = 2.4, df = 332, p < 0.02) (Figure 2).

## Discussion

We found that indeed, a significant improvement of playing ability was only seen in the MHD group, while in the ED group, there was no significant change of playing ability; this confirmed our 1^st^ hypothesis. Moreover, participants in the ED group rated their playing ability significantly lower than those in the MHD group both at onset of dystonia and at the timepoint when filling in the questionnaire, thus confirming our 2^nd^ hypothesis. Also, the difference in playing ability (today minus at onset) was significantly higher for the MHD group and virtually non-existent for the ED group, thus confirming hypothesis 3.

Finally, significantly fewer ED-patients had undergone therapies, most likely reflecting the fewer options compared to MHD-patients.

The lower playing ability in ED at onset as well as the lower amount of improvement in the course of ED reflects the fact that in ED, the sound production itself is impaired often making the production of a specific tone-register impossible [18] and leading to unstable sound that is not compatible with professional requirements [19]. In this context, it is important to recognize the immense demands placed on brass and woodwind players by the complex embouchure technique. The temporal-spatial coordination and interplay of tension and flexibility in movement patterns — including synchronization of the facial muscles, oral cavity muscles, tongue, pharynx, vocal apparatus, diaphragm and respiratory muscles — is extremely challenging. Furthermore, the strength and stamina required to play long “forte” notes, especially in the high registers of brass instruments, places enormous physical demands on players.

Additionally, wind instrumentalists play solo roles in orchestras, meaning that any reduction in sound quality is clearly audible. Therefore, the means for compensation are more limited in ED than in MHD. In string-instrumentalists e.g., the sound production, quality and differentiation can still be maintained by the right hand if the left hand is affected. Different fingerings may then be applied to avoid the affected fingers. The same is true for keyboard players, where changing the fingering can compensate to a certain degree for the difficulties of the more affected finger. The unaffected fingers (and the healthy side) would still be able to produce the desired timing and sound quality, for which moreover more proximal and unaffected muscles are involved as well [20,21]. In addition, many patients report that they adapt their musical repertoire by playing only pieces which are playable with dystonia. The composer Robert Schumann adapted his early piano works to his involuntary dystonic movements of the right middle fingers by avoiding fingerings which required the use of his affected right middle finger on black keys [22]. Jazz pianists may compensate by different improvisatory styles for difficulties emerging by involuntary finger movements. One study even found that patients with MHD tend to “positively overestimate the development of their symptoms” [23]. This was explained by the application of the compensatory mechanisms discussed above.

Furthermore there is a reduced amount of therapeutic options for ED patients: BTX being ineffective or even unfeasible as it would be necessary, among others, to determine the precise muscle areas of the tongue that are involved and administer BTX injections to target these areas [4,5,7,8]; also, THX has been rated as less efficient [4]. With regard to the retraining-therapy, again it is more difficult in ED than in MHD to first identify the affected of the more than 12 muscles involved in the embouchure [7] and then to address these specific muscles. Notably, there are several studies on retraining therapy for MHD [24,25,26], whereas retraining for ED has not been the focus of scientific research to date, possibly due to the difficulties mentioned above.

Due to these factors, ED is feared by wind players as a career-ending condition. This can lead to the problem being acknowledged too late, resulting in delayed consultation with a medical expert and delayed treatment. This can also have an adverse effect on the prognosis, as early treatment generally has a positive impact on the outcome.

We are aware that the interpretation of these results must be undertaken cautiously, since a retrospective study is prone to recall bias. Other limitations of the study include the lack of objective symptom severity measurements and the fact that we were unable to establish an association between symptom severity and individual therapeutic strategies due to the retrospective nature of the study. However, this is so far the largest sample size comparing MHD and ED patients. Together, our present findings show that the reduced improvement in ED patients compared to MHD patients are likely due to the limited therapeutic options as well as the limited means for compensating for the disorder, since the sound production itself is impaired.

Thus, there is an urgent need for alternative therapeutic measures, especially for patients suffering from ED to improve their relatively poor prognosis; especially retraining programs should be developed especially for this movement disorder, as retraining therapy has been shown to be quite effective in other task specific dystonias in musicians [4]. Moreover, given the immense demands placed on wind players by the complex embouchure technique, which makes them particularly vulnerable to ED, the importance of prevention in the field of music education should be recognized and promoted even more strongly.
